# Clinical implications of myeloid malignancy‑related somatic mutations in aplastic anemia

**DOI:** 10.1007/s10238-023-01067-4

**Published:** 2023-04-22

**Authors:** Lingling Liu, Danfeng Zhang, Qiuhao Fu, Jingdi Wang, Jifeng Yu, Dandan Chen, Fang Wang, Rong Guo, Xinsheng Xie, Zhongxing Jiang, Yingmei Li

**Affiliations:** Department of Hematology, The First Affiliated Hospital of Zhengzhou University, #1 Jianshe East Road, Zhengzhou, 450000 People’s Republic of China

**Keywords:** Aplastic anemia, Somatic mutations, Next generation sequencing, Clinical outcome

## Abstract

Aplastic anemia (AA) is a potentially fatal bone marrow failure syndrome characterized by a paucity of hematopoietic stem cells and progenitor cells with varying degrees of cytopenia and fatty infiltration of the bone marrow space. Recent advances in genomics have uncovered a link between somatic mutations and myeloid cancer in AA patients. At present, the impact of these mutations on AA patients remains uncertain. We retrospectively investigated 279 AA patients and 174 patients with myelodysplastic syndromes (MDS) and performed targeted sequencing of 22 genes on their bone marrow cells using next-generation sequencing (NGS). Associations of somatic mutations with prognostic relevance and response to treatment were analyzed. Of 279 AA patients, 25 (9.0%) patients had somatic mutations, and 20 (7.2%) patients had one mutation. The most frequently mutated genes were ASXL1(3.2% of the patients), DNMT3A (1.8%) and TET2 (1.8%). In the MDS group, somatic mutations were detected in 120 of 174 (69.0%) patients, and 81 patients (46.6%) had more than one mutation. The most frequently mutated genes were U2AF1 (24.7% of the patients), ASXL1 (18.4%) and TP53 (13.2%). Compared with MDS patients, AA patients had a significantly lower frequency of somatic mutations and mostly one mutation. Similarly, the median variant allele frequency was lower in AA patients than in MDS patients (6.9% vs. 28.4%). The overall response of 3 and 6 months in the somatic mutation (SM) group was 37.5% and 66.7%, respectively. Moreover, there was no significant difference compared with the no somatic mutation (N-SM) group. During the 2-years follow-up period, four (20%) deaths occurred in the SM group and 40 (18.1%) in the N-SM group, with no significant difference in overall survival and event-free survival between the two groups. Our data indicated that myeloid tumor-associated somatic mutations in AA patients were detected in only a minority of patients by NGS. AA and MDS patients had different gene mutation patterns. The somatic mutations in patients with AA were characterized by lower mutation frequency, mostly one mutation, and lower median allelic burden of mutations than MDS. Somatic mutations were a common finding in the elderly, and the frequency of mutations increases with age. The platelet count affected the treatment response at 3 months, and ferritin level affected the outcome at 6 months, while somatic mutations were not associated with treatment response or long-term survival. However, our cohort of patients with the mutation was small; this result needs to be further confirmed with large patient sample.

**Supplementary Information:**

The online version contains supplementary material available at 10.1007/s10238-023-01067-4.

## Introduction

Acquired aplastic anemia (AA) is the prototype of the bone marrow failure syndrome characterized by hypocellular marrow and pancytopenia. The disease is typically acquired, and the principal mechanism is that activated T cells produce proinflammatory cytokines and proteins that are associated with a lack of immune regulation and recognize and eliminate hematopoietic stem or progenitor cells [1–3]. The main treatment methods are transplantation and immunosuppressive therapy (IST). Bone marrow transplantation is curative, and patients may have a better response rate to standard immunosuppressive therapy combined with eltrombopag [2, 4]. Long-term follow-up has shown that about 10–20% of AA patients evolved to myelodysplastic syndromes (MDS) or acute myeloid leukemia (AML), especially who did not achieve a complete response following treatment with immunosuppressive therapy [5, 6].

With the advent of next-generation sequencing (NGS), the presence of acquired somatic mutations in myeloid candidate genes previously associated with MDS/AML emerged as a common finding in patients with AA. Prior studies reported that mutations in *PIGA* and *BCOR* had a better response to immunosuppressive therapy and improved clinical outcome, specifically improved progression‐free survival (PFS) and overall survival (OS), while AA patients with mutated *ASXL1*, *DNMT3A*, *JAK2/JAK3*, or *RUNX1* fare worse with regard to immunosuppressive therapy response and survival [7, 8]. A high-risk clonal evolution was detected in AA patients with *ASXL1*, *DNMT3A* or *RUNX1* mutations [8–10]. However, recent studies demonstrated that mutations did not correlate with hematologic response and OS, either at baseline or in new or additional mutations; treating physicians should not overinterpret the presence of mutant clones at diagnosis or after therapy in isolation, and detection of these clones do not warrant high-risk procedures, such as hematopoietic stem-cell transplantation [2, 11].

Thus far, the impact of somatic mutations in AA on clinical and hematological outcomes is not completely established. Therefore, we conducted this study using bone marrow samples of AA patients in targeted sequencing to investigate the frequencies of somatic mutations in AA and their correlation with prognostic relevance and response to immunosuppressive treatment.

## Materials and methods

### Patients

We investigated a cohort of 279 hospitalized AA patients from the hematology department of the First Affiliated Hospital of Zhengzhou University between January 2016 and June 2021. At the time of enrollment, we excluded patients without completion of next-generation sequencing. The following laboratory and clinical information were obtained for each patient: blood cell count, ferritin, iron and the somatic mutations of bone marrow cells, age, sex, date of diagnosis and treatment. Furthermore, patients were followed up till September 2022 for disease progression, survival and response to treatment. AA patients were diagnosed according to 2016 British criteria, and the inherited AAs were excluded. [12] The severity of AA was graded according to the blood count parameters and bone marrow findings. Severe AA (SAA) was defined as BM cellularity < 25%, or 25–50% with < 30% residual hematopoietic cells and at least two of the following: (I) absolute neutrophil count < 0.5 × 10^9^/L, (II) platelets < 20 × 10^9^/L and (III) reticulocyte count < 20 × 10^9^/L. AA patients who did not meet the criteria for SAA were classified as non-severe AA (NSAA). The treatment outcome was based on previous literature [4, 11, 13]. Overall survival (OS) was defined as the time from enrollment to death from any cause or the last follow-up. Event-free survival (EFS) was defined as the first event time from diagnosis to the last follow-up. Event: clonal evolution (MDS/AML), relapse, death, and progression to severe AA. For comparison, we included a cohort of 174 MDS patients who performed targeted sequencing during the same period, diagnosed according to the 2016 World Health Organization classification [14]. To better compare with AA patients, we screened MDS from patients with pancytopenia.

The inclusion criteria were: (1) age ≥ 14 years old, (2) NGS was performed and (3) complete clinical data.

### Gene sequencing

Gene mutation analysis was performed by NGS of the DNA samples extracted from the bone marrow monocytes in patients. The NGS libraries were paired-end sequenced (2 × 150 bp) on an Illumina MiSeq System (Illumina, San Diego, CA). The mean depth of each sample was 2500 × , with an average of 98% of the target sequence covered sufficiently deep for variant calling. Detection sensitivity was ~ 5% (a mutation with 5% or more variability can be reported).

The Illumina MiSeq System (Illumina, San Diego, CA) is a high-throughput sequencing platform based on Sequencing by Synthesis (SBS) technology and sequence libraries to produce large amounts of high-quality data. Analyses were conducted of the relevant mutations of 22 genes, *ASXL1*, *NPM1*, *KIT*, *FLT3*, *CEBPA*, *DNMT3A*, *IDH1/2*, *TET2*, *EZH2*, *RUNX1*, *PHF6*, *TP53*, *SF3B1*, *SRSF2*, *U2AF1*, *ZRSR2*, *NRAS*, *CBL*, *SETBP1*, *ETV6*, and *JAK2*. Each mutation was analyzed in the Catalog of Somatic Mutations in Cancer databases (COMSIC; https://cancer.sanger.ac.uk/cosmic/). All bone marrow samples were collected with informed consent, and the study was reviewed and approved by the First Affiliated Hospital of Zhengzhou University College of Medicine.

### Statistical analysis

Continuous variables were described by median and range, while classification variables were described by example and percentage. Mann–Whitney U test was used for continuous variables, and the chi-square test and Fisher exact test were used for classified variables. The OS and EFS were estimated using the Kaplan–Meier curve and compared by the Log-rank test. The logistic regression model was used for single and multivariate analyses, and the variables with *P* < 0.1 were included in the multivariate analysis. The statistical analyses were performed using SPSS version 20.0. GraphPad Prism 8 was run to generate graphs. A *P*-value of < 0.05 was considered statistically significant.

## Results

### Somatic mutation

**Fig. 1:**
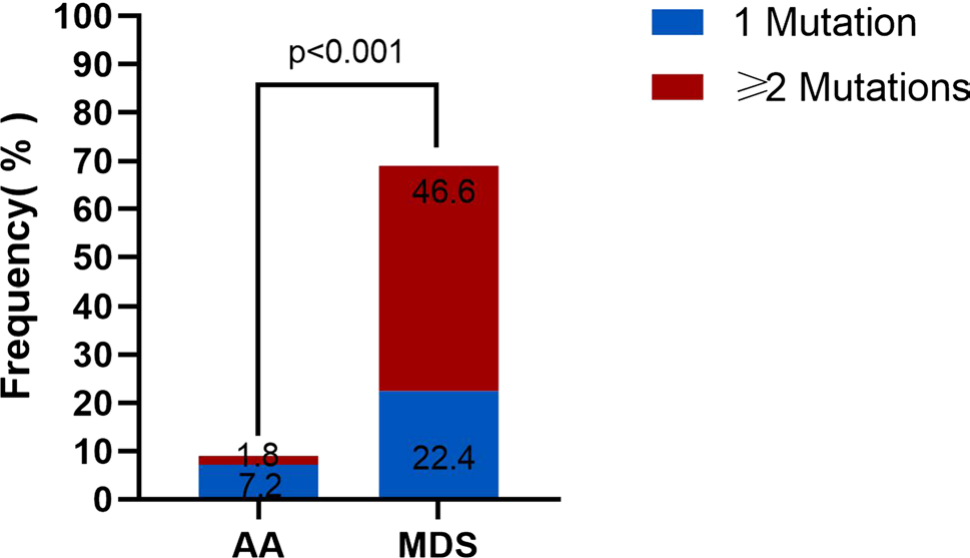
The bar chart shows the frequency and number of mutations in AA and MDS. Blue color indicates the frequency of patients with one mutation (7.2% in AA or 22.4% in MDS), blue indicates the frequency of patients with two or more mutation (1.8% in AA or 46.6% in MDS)

**Fig. 2:**
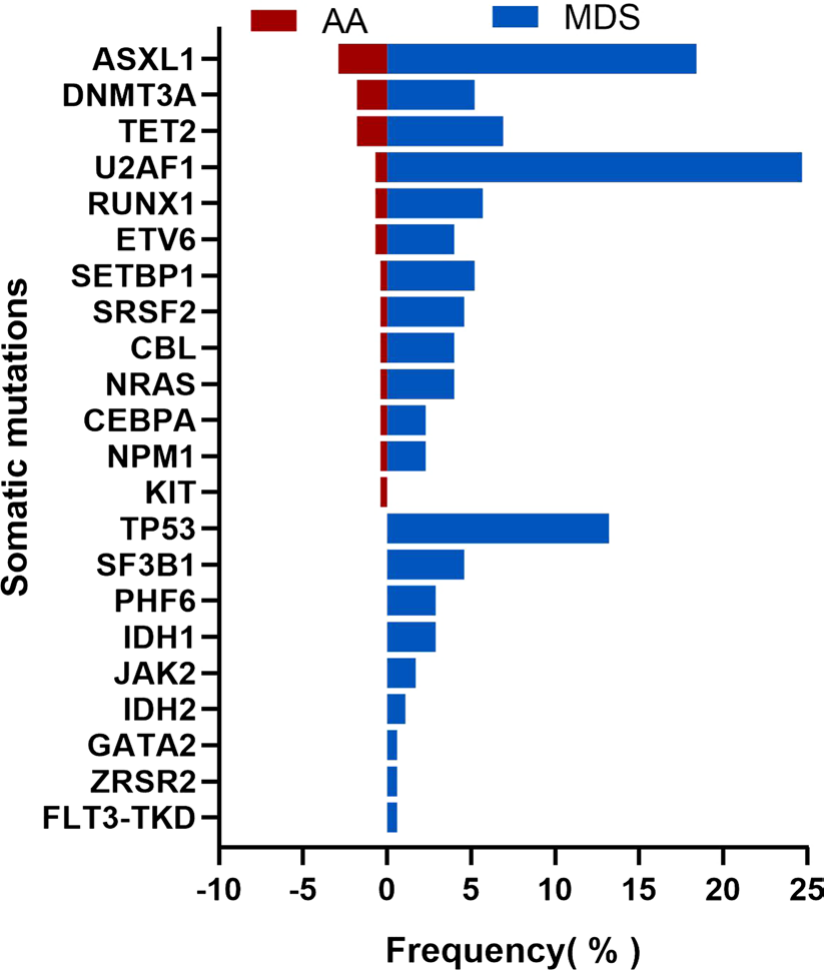
Bar chart showing the frequency of mutated genes and type of mutations in each gene identified in the AA and MDS groups

**Fig. 3:**
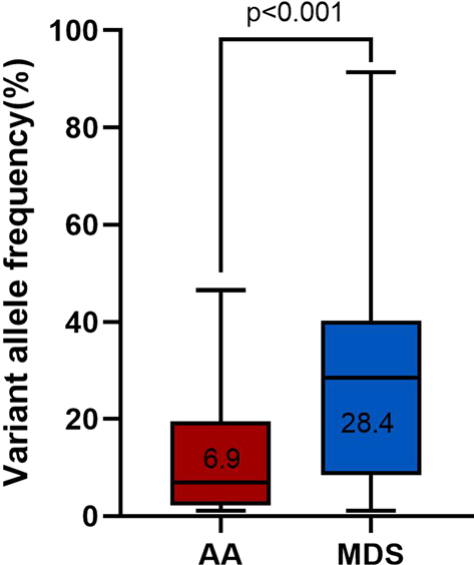
Box figure show the median variant allele frequency in the AA and MDS cohorts. The box-and-whisker plots of the specific gene mutations are shown; the whiskers indicate the range, the sides of the boxes indicate the interquartile range, and the vertical line within each box indicates the median(6.9% vs. 28.4%). *P* values were obtained using Wilcoxon rank sum test

**Fig. 4:**
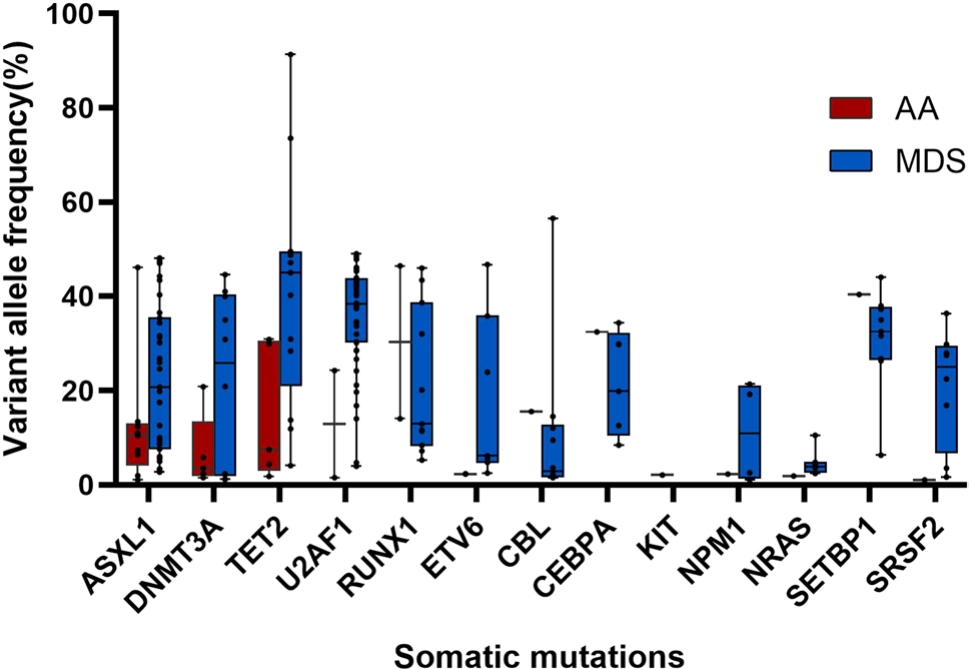
Box figure show the variant allele frequency of individual mutations in the AA and MDS cohorts. The box-and-whisker plots of the specific gene mutations are shown; the whiskers indicate the range, the sides of the boxes indicate the interquartile range, and the horizontal line within each box indicates the median

A total of 279 AA patients were enrolled in this study, including 128 females (45.9%) and 151 males (54.1%), with a median age of 39(14–85) years at diagnosis. Targeted NGS of a panel of genes that were recurrently mutated in myeloid cancers was performed using bone marrow obtained from the patients. Overall, there were 25 patients (9.0%) with somatic mutations, 20 patients (7.2%) had one mutation, four patients (1.4%) had two mutations, and one patient (0.4%) had more than two mutations (Fig. 1 Frequency of Mutation in the AA and MDS groups). The most frequently mutated genes were *ASXL1*(in 2.9% of the patients), *DNMT3A* (1.8%) and *TET2* (1.8%) (Fig. 2 Somatic Mutations Identified by next-generation sequencing and Supplementary Table 1). Details of the somatic mutations in AA patients are given in Supplementary Table 2. Moreover, there were 174 MDS patients, 112 males (64.4%), and the median age at diagnosis was 55 (15–87) years. Analysis of mutation results, at least one gene mutation was detected in 120 patients (69.0%), including 39 patients (22.4%) who had one mutation, 48 patients (27.6%) had two mutations, and 33 patients (18.9%) had more than two mutations (Fig. 1). The most frequently mutated genes were *U2AF1* (in 24.7% of the patients), *ASXL1* (18.4%) and *TP53* (13.2%) (Fig. 2 and Supplementary Table 1). The number of mutations per patient was lower in AA than in MDS, typically one mutation per patient compared with a median number of two in MDS. There was a statistically significant difference in the number of mutations between the two groups (Fig. 1). At the same time, the gene type and frequency of each gene mutation type were fewer than in the MDS group (Fig. 2 and Supplementary Table 1). Furthermore, the median variant allele frequency (VAF) in AA was substantially lower than that of patients with MDS (6.9% vs. 28.4%) with statistical significance (Fig. 3 Comparison of the median variant allele frequency between AA and MDS cohorts). Similarly, the median VAF of each somatic mutation was lower in AA patients than MDS, as shown in Fig. 4 (Comparison of variant allele frequencies individual mutation between the AA and MDS cohorts). A comparison of the mutation of patients in various age groups showed that the proportion of mutation in MDS patients increased with age. The incidence of gene mutation in the AA group increased with the trend, but there was no statistical significance (Supplementary Fig. 1 Prevalence of somatic mutations, according to age).

### Clinical correlations

Patients were classified into two groups by mutation: 25 patients with a somatic mutation (SM, 9.0%) and 254 patients with no somatic mutation (N-SM, 91.0%). In the SM group, the median age was 53 (14–85) years, significantly higher than the N-SM group, with a median age of 38 (14–77) years (*P* < 0.05, Table 1). Among 279 patients with AA, we excluded five patients who had transplant within 3 months, and 29 patients were lost to follow-up. Finally, we enrolled 245 patients to assess treatment response, prognosis and the relationship between somatic mutation, consisting of the SM group (*n* = 24, 9.8%) and the N-SM group (*n* = 221, 90.2%). The median age of the patients in the SM group was higher than in the N-SM group (*P* = 0.012). Comparing laboratory examination, we found that the SM group had a higher monocyte count than the N-SM group, with the values of 0.18 (0.02–0.89) × 10^9^/L vs. 0.13 (0.00–0.75) × 10^9^/L. There was a statistically significant difference between the two groups (*P* < 0.05, Table 2). However, other blood parameters were not significantly different between SM patients and N-SM patients. There were no significant differences between patients with or without mutations in the severity of AA (*P* = 0.785), cytogenetics (*P* = 0.164) and treatment (*P* = 0.564).

**Table 1:** Association analysis of clinical characteristics with presence or absence of somatic mutation in AA

Characteristic	*n*	Somatic mutation	No somatic mutation	*P* value
Sex— no. (%)				0.520
Male	151	12(7.9)	139(92.1)	
Female	128	13(10.2)	115(89.8)	
Age, years				0.040
Median	–	53	38	
Range	–	14–85	14–77	
Severity of AA— no. (%)				0.785
NSAA	149	14(9.4)	135(90.6)	
SAA	130	11(8.5)	119(91.5)	
Cytogenetic— no. (%)				0.164
Normal	189	19(100)	170(98.3)	
Abnormal	3	0(0)	3(1.7)	
Treatment (%) ※				0.764
ATG + CSA/TPORA	26	2(8.3)	24(10.9)	
CSA + /TPORA	184	20(83.3)	164(74.2)	
Others	35	2(8.3)	33(14.9)	
Response at 3 Mo (%) ※				0.660
OR	82	9(37.5)	73(33.0)	
NR	163	15(62.5)	148(67.0)	
Response at 6 Mo (%) *				0.052
OR	106	14(66.7)	92(44.4)	
NR	121	7(33.3)	115(55.6)	

*NSAA* non-aplastic anemia, *SAA* severe aplastic anemia, *VSAA*, very severe aplastic anemia, *OR* (overall response), *CR* (complete response) + PR (part response), *NR* no response *EFS* event-free survival

※5 transplant patients within 3 months and 29 patients lost to follow-up, were excluded in this analysis

*11 transplant patients within 6 months, 29 patients lost to follow-up, and 9 died within 3 months of the patients, were excluded in this analysis

**Table 2:** Association analysis of laboratory examination with presence or absence of somatic mutation in AA

	Somatic mutation	No somatic mutation	*P* value
WBC	2.28(0.95–3.60)	2.10(0.20–3.90)	0.585
Hb(g/L)	66(22–88)	65(23–109)	0.822
Ret(× 10^9^/L)	25.9(2.3–74.9)	29.6(2.2–87.0)	0.901
ANC(× 10^9^/L)	0.71(0.01–1.49)	0.62(0.02–2.33)	0.299
Ly(× 10^9^/L)	1.39(0.51–2.32)	1.22(0.06–3.13)	0.585
Mo(× 10^9^/L)	0.18(0.02–0.89)	0.13(0.00–0.75)	**0.021**
PLT(× 10^9^/L)	11(4–49)	12(1–59)	0.701
RDW (%)	15.8(12.5–23.6)	15.1(10.7–20.9)	0.315
Iron (umol/L)	40.2(9.1–68.1)	36.8(7.1–74.8)	0.740
Ferritin (ug/L)	546 (121.4–3405.7)	576.0(8.5–5916.1)	0.597

Bold indicates that the *P*-value is statistically significant

Median (range). *Hb* Hemoglobin, *Ret* Reticulocyte, *ANC* absolute neutrophil count, *Ly* Lymphocyte count, *Mo* monocyte, *PLT* platelet, *RDW* Red blood cell distribution width

AA patients were subdivided into three groups according to treatment: anti-thymocyte globulin combined with or without cyclosporine/thrombopoietin-receptor agonist (Eltrombopag /Herombopag), cyclosporine combined with or without thrombopoietin-receptor agonist (Eltrombopag /Herombopag), and others (supportive or other immunosuppressive therapy). Furthermore, we included the efficacy of treated AA patients for three and 6 months to assess the potential impact of somatic mutations on the treatment response. In the SM group, the overall response to IST at three and 6 months were 37.5% and 66.7%, respectively, and there was no significant difference compared with the N-SM group (*P* > 0.05, Table 1). Additionally, we included the factors that might affect treatment in logistic, univariate and multivariate analyses. The results showed that mutation, reticulocyte, absolute neutrophil count and lymphocyte count did not affect the efficacy(*P* > 0.05,); only the platelet count and the treatment options affected the treatment response at 3 months (*P* < 0.05, Tables 3, 4). Surprisingly, ferritin influenced the 3-month overall response of patients in univariate analysis. Similar results were found for the influence factor of overall response at 6 months (*P* < 0.05, Table 3).

**Table 3:** Univariate and multivariable analysis of predictors affecting OR at (A) 3 months and (B) 6 months

variables	Univariate analysis		Multivariable analysis	
	HR (95%CI)	*P* value	HR (95%CI)	*P* value
(A)				
Years	0.99(0.98–1.01)	0.222	0.39(0.13–1.13)	0.202
Disease severity	1.30(0.76–2.22)	0.344	0.99(0.97–1.01)	0.083
Mutations	1.22(0.51–2.91)	0.660	2.18(0.70–6.81)	0.181
PNH	0.91(0.64–1.30)	0.607	1.26(0.55–2.86)	0.588
ANC(× 10^9^/L)	0.87(0.47–1.58)	0.64	0.57(0.17–1.86)	0.350
Ly(× 10^9^/L)	0.95(0.65–1.51)	0.95	0.83(0.47–1.44)	0.500
PLT(× 10^9^/L)	1.02(1.00–1.05)	**0.044**	1.05(1.01–1.08)	**0.013**
Ret(× 10^9^/L)	1.00(0.99–1.02)	0.590	0.99(0.98–1.01)	0.415
Ferritin (ug/L)	1(0.99–1.00)	**0.031**	1(0.99–1.00)	0.387
Treatment	5.64(1.75–18.16)	**0.004**	10.76(2.07–55.91)	**0.005**
(B)				
Years	0.99(0.98–1.01)	0.634	0.99(0.97–1.01)	0.610
Disease severity	1.25(0.73–2.12)	0.403	1.40(0.48–4.11)	0.536
Mutations	2.44(0.94–6.29)	0.066	2.87(0.96–8.58)	0.059
PLT(× 109/L)	1.01(0.99–1.04)	0.255	1.02(0.99–1.05)	0.328
Ferritin (ug/L)	1(0.99–1.00)	**0.013**	1.00(0.99–1.00)	**0.035**
Treatment	0.29(0.10–0.82)	**0.015**	4.75(1.07–21.05)	**0.040**

Bold indicates that the *P*-value is statistically significant

OR(overall response), CR (complete response) + PR (part response); Disease severity, non- severe aplastic anemia versus severe aplastic anemia; PNH, Paroxysmal nocturnal hemoglobinuria with cutoff > 1%; PLT, Platelet. For all multivariable modeling, a series of factors were pre-identified as possibly prognostic: age; disease severity (NSAA vs. SAA and NSAA, SAA vs. VSAA); PNH clone at baseline as assessed by flow cytometry (> 1% versus ≤ 1%); treatment. Additionally, hemoglobin, neutrophils, absolute lymphocytes and reticulocytes counts were considered

**Table 4:** Therapeutic response analysis of ASXL1/DNMT3A somatic mutation in AA

	*n*	Somatic mutation	No somatic mutation	*P* value
Response at 3 Mo (%)				0.764
OR	78	5(38.5)	73(33.0)	
NR	156	8(61.5)	148(67.0)	
Response at 6 Mo (%)				0.118
OR	100	8(72.7)	92(45.1)	
NR	115	3(27.3)	112(54.9)	

OR(overall response), CR (complete response) + PR (part response); NR, no response

### Longer-term follow-up

**Fig. 5:**
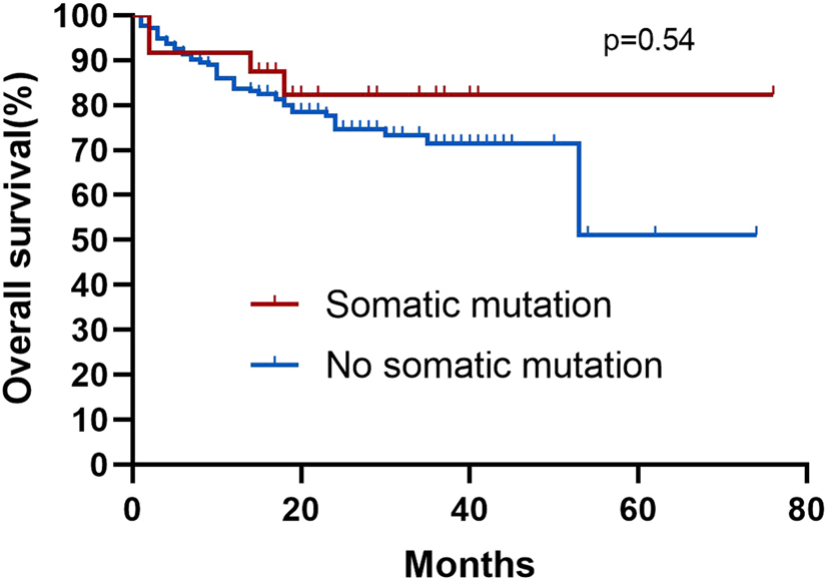
Kaplan–Meier curves for overall survival among patients with and without somatic mutation. Log-rank tests were used for statistical comparisons among the groups(*p* = 0.54)

**Fig. 6:**
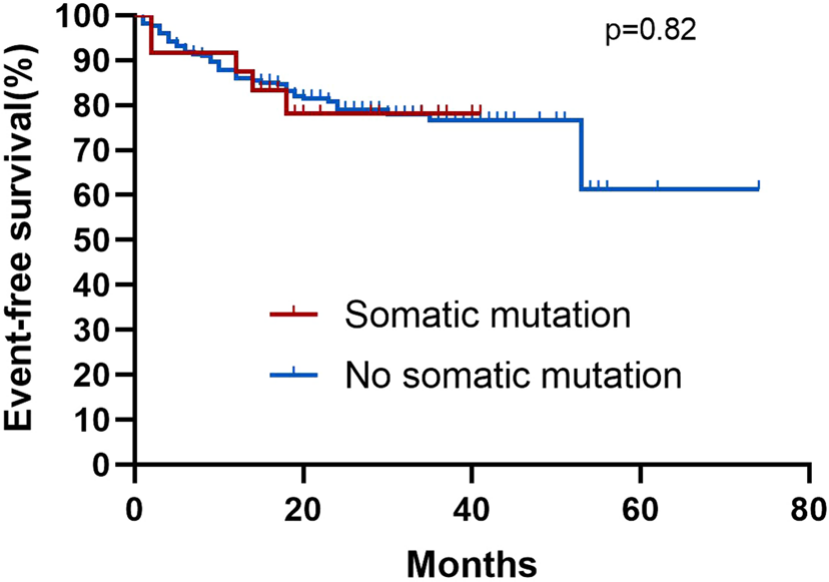
Kaplan–Meier curves for event-free survival among patients with and without somatic mutation. Log-rank tests were used for statistical comparisons among the groups (*p* = 0.82)

**Fig. 7:**
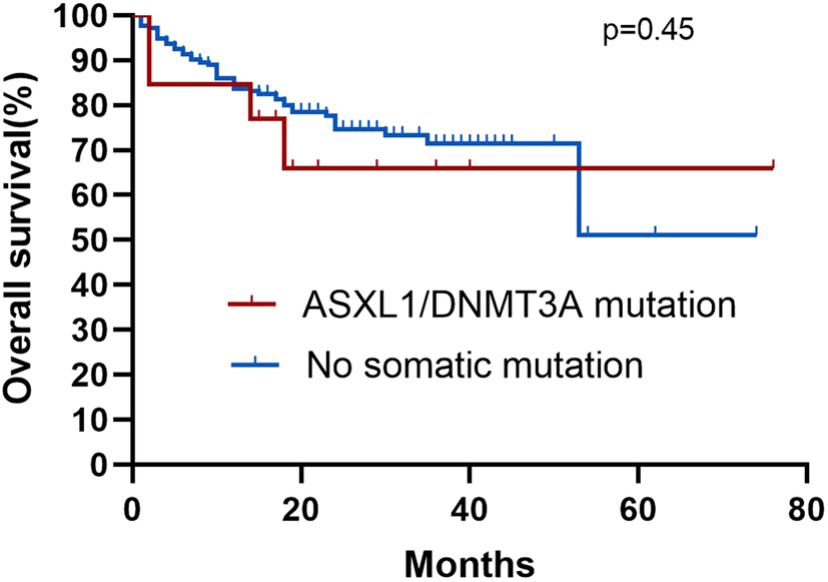
Kaplan–Meier curves for overall survival among patients with and without ASXL1/DNMT3A somatic mutation. Log-rank tests were used for statistical comparisons among the groups (*p* = 0.45)

**Fig. 8:**
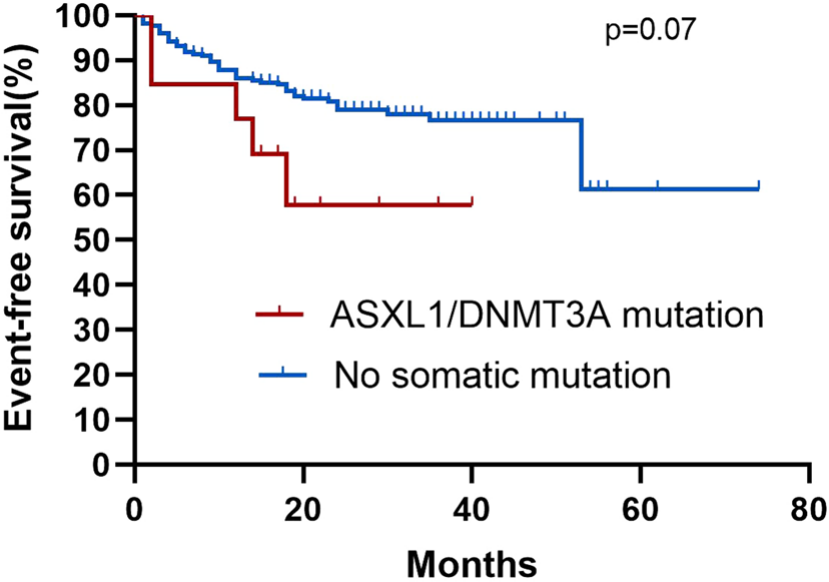
Kaplan–Meier curves for event-free survival among patients with and without ASXL1/DNMT3A somatic mutation. Log-rank tests were used for statistical comparisons among the groups (*p* = 0.07)

Among the 245 AA patients, the median follow-up was 25 months (95% confidence interval [CI] 25.3–28.8) in all patients. The median follow-up among the patients in SM and N-SM groups was 22 months (95% CI 19.2–32.1) and 26 months (95% CI 25.4–29.0). During the entire follow-up period, four (16.7%) patients in the SM group and 44 (19.9%) patients in the N-SM group died. By comparing the survival curves of the mutant and non-mutant groups, we found that the presence of somatic mutations had no significant effect on the AA patients OS (Fig. 5 Overall survival correlations with somatic mutations). In the SM group, five (20.8%) patients had an event, while 48 (21.7%) patients had an event in the N-SM group, Kaplan–Meier curves for EFS were not significantly different (Fig. 6 Event-free survival correlations with somatic mutations). At the same time, we also compared the OS and EFS of *ASXL1* and *DNMT3A* mutant and non-mutated groups, which was not statistically significant (Fig. 7 Overall survival correlations with *ASXL1/DNMT3A* somatic mutation and Fig. 8 Event-free survival correlations with *ASXL1/DNMT3A* somatic mutation).

Three patients who were older than 50 years had clonal progression to MDS at four, 4, and 9 months, respectively, after immunosuppressive therapy. Moreover, there were three cases of secondary MDS patients without somatic mutation before transformation and the normal chromosome karyotype. Two had no response at 6 months, while the third had a partial response. After transformation, one patient had a karyotype change of 46XX, − 7 (− 7/7q−), and one patient had *ASXL1* mutation, both of them died at 9 and 17 months, respectively. One patient carrying *ASXL1*, *SETBP1* and *RUNX1* mutations evolved to AML within seven years of the AA diagnosis; this patient was diagnosed with NSAA at the age of 47 and received oral cyclosporine therapy for two years, but the disease relapsed after discontinuation of the drug. Moreover, there was no durable response upon subsequent cycles of immunosuppressive therapy. The cytogenetic karyotype at the time of evolution was 46XY, − 7, + 21[7]/46XY [3].

## Discussion

With the advancement of NGS, somatic mutations associated with myeloid malignancies have been found in AA patients. Somatic mutations are associated with clonal hematopoiesis in AA, affecting the treatment and survival and further clonal evolution into MDS/AML. In the present study, 279 AA patients were included, and MDS was used as a control to clarify the proportion of somatic mutations in AA patients in our hospital. In addition, we followed up with 245 patients with AA, analyzing the influence of the somatic mutation on treatment and survival to help doctors make treatment decisions.

In our study, 9% of AA patients had myeloid tumor-related somatic mutations. Similarly, Daria V Babushok et al. reported mutations only in a small subset of patients with AA (9%), and Heuser M et al. identified three somatic mutations in two patients in the examined MDS candidate genes (5.3%). [15, 16] However, compared with most studies that reported somatic mutations in about 21% of patients, mutation frequency in our study was significantly lower, which could be due to the small number of gene panel for targeted sequencing and the absence of the most common mutated genes in AA: *PIGA* and *BCOR*. [7, 8, 10, 17, 18] The most commonly mutated genes in AA were *ASXL1*, *DNMT3A* and *TET2*. After excluding *PIGA* and *BCOR* mutations, the most frequently mutated genes were *ASXL1, DNMT3A* and *TET2* in AA patients, similar to the results of our study. [10, 19, 20] The mutation pattern in AA patients were different from the MDS patients. In our study, the most frequently mutated genes in MDS patients were *U2AF1*, *ASXL1* and *TP53*. It is worth noting that *SF3B1* mutation frequency was pretty low compared with previous studies [21]. The presumed reasons are as follows. First, *SF3B1* mutations were mainly found in MDS patients with ring sideroblasts, and the hemogram of these patients generally presented decreased hemoglobin [22]. We excluded patients with anemia alone, which may lead to a lower SF3B1 mutation in the MDS cohort. Second, it may be due to the small sample size of our study. Although *ASXL1* and *DNMT3A* mutations were frequent in AA, MDS and AML, there is a substantial underrepresentation of mutations in splicing-factor genes, *JAK2*, and *TP53* in AA compared with MDS and AML, reflecting the difference in the mechanism of clonal selection between both diseases and mutations. [3, 23–25] Meanwhile, our data showed that the median allelic burden of mutations in AA was substantially lower than that in MDS (6.9% vs. 28.4%). The low-burden mutations might be transient events and might not contribute to later evolution. However, along with somatic chromosomal aberrations, the mutational burden (total volume of acquired mutations) might serve as a measure of genomic damage. [26]

Benign clonal hematopoiesis identified in healthy individuals is known as clonal hematopoiesis of indeterminate potential(CHIP). The somatic mutations that drive CHIP were most frequently involved in *DNMT3A* and *TET2*, which are also more frequent in AA. The difference in somatic mutations between AA patients and CHIP lesion populations remains to be explored. *DNMT3A* and *TET2* mutations in AA patients might represent CHIP lesions. In AA, somatic mutations become detectable as the contraction of the stem cell compartment leads to decreased hematopoietic stem cells. However, concerning previous literature, we have different speculation. Research showed that the frequency of mutations increases in frequency with age in the general population. Mutations in genes implicated in hematologic cancers were found in < 1% of healthy persons younger than 40 years of age, 1.7% of persons 40–49 years of age, 2.5% of persons 50–59 years of age, 45.2% of persons 60 years of age or older [27]. The mutations in *DNMT3A* and *TET2* comprised 57.2% and 33.3% of all recorded mutations in CHIP lesions, respectively [28]. Furthermore, the prevalence of *TET2* mutations in individuals 55 years of age or older showed a consistent increase with age, averaging 6.8% per year [29]. In our study, somatic mutations were observed in 5.9% of patients younger than age 40 years, 12.2% in those 40–49 years, 16.7% in those 50–59 years, and 24.8% of patients 60 years of age or older. *DNMT3A* and *TET2* mutations accounted for the same proportion of all documented mutations in AA patients, which was 16.1% (5/31). Moreover, when the patient was younger than 60 years old, the frequency of somatic mutations in AA patients in our study was higher than that in previous studies of CHIP lesions. Additionally, the mutation frequencies of *DNMT3A* and *TET2* were different in AA and CHIP patients. Finally, although somatic mutations did not affect the treatment response and survival of AA patients in our study, Huang et al. [8] reported that AA patients with *TET2* mutations had a better response to IST than unmutated ones, and Park et al. [18] suggested *DNMT3A* mutations was an adverse factor associated with short overall survival. In conclusion, we speculate that the *DNMT3A* and *TET2* mutations in AA patients may be different from those in CHIP lesions, but further follow-up is needed for verification.

The somatic mutations were a common finding in the elderly [30]. According to our data, SM patients were older than N-SM patients, but the mutation frequency does not increase markedly with age. On the one hand, it is possible that with aging, there is a stereotypical outgrowth of cells bearing mutations in epigenetic regulators of stem cell renewal, *DNMT3A*, *TET2*, and *ASXL1*, as well as others [31]. This age-dependent background of stochastic mutations also shapes clonal diversity in AA, as hematopoietic and progenitor cells with various background mutations “struggle for existence” and may derive a selective advantage from their somatic differences [17]. Indeed, AA cases presenting at an older age may already carry somatic age-related mutations, which could be positively selected after multiple rounds of IST because of the inherent increased fitness advantage of oligoclonal over normal hematopoietic stem and progenitor cells.[20] On the other hand, it is considered that somatic mutations may occur during the pathogenesis of AA. The depletion of AA stem cell pool was conducive to the growth of some hematopoietic stem cells with somatic mutations. These hematopoietic stem cells through acquisition of somatic alterations, becomes either less immunogenic or acquires relative resistance to cytotoxicity or cytokine-mediated marrow suppression, were more likely to survive [32, 33]. Babushok et al. showed that clonal hematopoiesis can occur early in the course of the disease even in young patients with AA, suggesting that somatic mutations in AA are not always associated with aging [15].

As for the relationship between somatic mutations and blood cell counts, studies have reported that red-cell distribution width is significantly increased in patients with somatic mutations and associated with all-cause death in patients. [27] At the same time, some studies indicated that AA patients with somatic mutations had higher neutrophil counts. [11] We analyzed the blood routine of the two groups and found that the monocyte count of the mutation patients was significantly higher, but there was no significant difference in red-cell distribution width and absolute neutrophil count between the two groups.

A long-term follow-up of 245 AA patients was conducted to observe the efficacy of IST treatment at 3 and 6 months. The results showed that somatic mutations did not affect the treatment response of AA patients. This is consistent with another research stating no significant differences in OS and response to IST between patients with somatic mutations and without mutations [10, 11, 34]. Previous studies reported that patients with *ASXL1/DNMT3A* mutations had poor immunosuppressive responses and shorter survival [7, 18]. In addition, we analyzed the response and survival of patients with *ASXL1* and *DNMT3A* mutations and without mutations, and the results showed no significant differences in both groups. Furthermore, new or additional mutations after treatment were not predictive of a lack of response or other dire outcomes. [11] Univariate and multivariate logistic regression displayed that the treatment regimen affected the response at three and 6 months. The somatic mutations and previously reported hematologic characteristics at baseline (absolute reticulocyte count and absolute lymphocyte count) were not associated with the overall response rate in our trial [35]. Surprisingly, platelet count affected the 3-month efficacy of AA patients, and ferritin influenced the 3-month overall response of patients in univariate analysis and the 6-month overall response.

Retrospective studies with long follow-up revealed that 10–20% of patients with AA treated with IST developed either MDS or AML, and patients of older age at diagnosis, a shorter telomere length, poor response to standard IST and longer disease duration were at higher risk of clonal evolution [5, 17, 20, 30]. Recently, in a cohort of 407 patients from the National Institutes of Health, the presence of specific somatic mutations was found to be predictive of evolution to MDS/AML when detected at 6 months after IST; including *RUNX1*, splicing factor mutations, and *ASXL1*.[9] By contrast, the predictive value of isolated mutations in genes like *TET2* and *DNMT3A*, which are frequently mutated in age-related clonal hematopoiesis was lower, these genes require additional genetic events to give rise to a myeloid neoplasm. [27, 36, 37] In our study, there were four cases of clonal progression; all were older than 50 years, three cases progressed to MDS with normal chromosomal karyotype and no detectable somatic mutations at the time of the sequence analysis before diagnosis, and one case progressed to AML with three somatic mutations, *ASXL1*, *SETBP1* and *RUNX1*, respectively. Overall, because of the rarity of AA and the long latency of clone evolution, the contribution of mutations to the risk of malignancy in AA patients is less clear, necessitating longer follow-up and larger patient numbers, and should be considered within the context of other patient and disease-specific factors, as well as the function of the mutated gene in hematopoiesis, aging, and autoimmunity [6, 34].

In conclusion, our data indicated that myeloid tumor-associated somatic mutations in AA patients were detected in only a minority of patients by NGS sequencing. AA and MDS patients had different gene mutation patterns. The somatic mutations in patients with AA were characterized by lower mutation frequency, mostly one mutation, and lower median allelic burden of mutations than MDS. Somatic mutations were a common finding in the elderly, and the frequency of mutations increases with age. The platelet count affected the response to treatment at 3 months, and ferritin level affected the outcome at 6 months, while somatic mutations were not associated with treatment response or long-term survival. Nevertheless, since our cohort of patients with the mutation was small, this result needs to be further confirmed with larger series of patients.

### Supplementary Information

Below is the link to the electronic supplementary material.

Supplementary file1 (DOCX 98 KB)
