# Supplementary material for: Clinical implications of myeloid malignancy‑related somatic mutations in aplastic anemia
Source: Clin Exp Med. 2023 Apr 22;23(8):4473–82. doi: 10.1007/s10238-023-01067-4 (PMC10725342; doi:10.1007/s10238-023-01067-4)
Supplement: Supplementary file 1 — Supplementary file1 (DOCX 98 KB) [file 10238_2023_1067_MOESM1_ESM.docx]

Table 1：Frequency of mutations in AA and MDS groups.

| Gene | AA | MDS | *P* value |
| --- | --- | --- | --- |
| ASXL1 | 2.9% | 18.4% | <0.001 |
| DNMT3A | 1.8% | 5.2% | 0.043 |
| TET2 | 1.8% | 6.9% | 0.005 |
| U2AF1 | 0.7% | 24.7% | <0.001 |
| RUNX1 | 0.7% | 5.7% | 0.002 |
| ETV6 | 0.7% | 4.0% | 0.031 |
| SETBP1 | 0.4% | 5.2% | 0.001 |
| SRSF2 | 0.4% | 4.6% | 0.003 |
| CBL | 0.4% | 4.0% | 0.006 |
| NRAS | 0.4% | 4.0% | 0.006 |
| CEBPA | 0.4% | 2.3% | 0.074 |
| NPM1 | 0.4% | 2.3% | 0.074 |
| KIT | 0.4% | 0.0% | - |
| TP53 | 0.0% | 13.2% | - |
| SF3B1 | 0.0% | 4.6% | - |
| IDH1 | 0.0% | 2.9% | - |
| PHF6 | 0.0% | 2.9% | - |
| JAK2 | 0.0% | 1.7% | - |
| GATA2 | 0.0% | 0.6% | - |
| IDH2 | 0.0% | 1.1% | - |
| FLT3-TKD | 0.0% | 0.6% | - |
| ZRSR2 | 0.0% | 0.6% | -^[[1]](#footnote-1)^ |
|  |  |  |  |

Table 2：Details of all the somatic mutations in AA patients.

| ID | somatic mutations | Protein | VAF (%) |
| --- | --- | --- | --- |
| 55 | ASXL1  SETBP1  RUNX1 | p. H630fs  p.E858K  p. Q185X | 7.38  40.34  46.44 |
| 214 | ASXL1 | p.R725fs | 46.06 |
| 19 | ASXL1 | p.Q780X | 12.4 |
| 136 | ASXL1 | p.Y700X  p.P1042fs | 11  10.5 |
| 14 | ASXL1 | p.G642fs | 6.5 |
| 79 | ASXL1 | p.G642fs | 13.46 |
| 231 | ASXL1 | p.D1004fs | 1.96 |
| 148 | ASXL1 | p.D1293fs | 1.1 |
| 252 | CBL  RUNX1 | p.C404Y  p.R201X | 15.59  14.08 |
| 53 | CEBPA  TET2 | p.P261fs  p.E1958fs | 32.42  30.8 |
| 188 | DNMT3A | p.L637Q | 5.85 |
| 196 | DNMT3A | p.Y735C | 1.55 |
| 139 | DNMT3A | p.R882S | 3.58 |
| 270 | DNMT3A | p.R882C | 2.36 |
| 87 | DNMT3A | p.R882H | 20.85 |
| 133 | ETV6  SRSF2 | p.R369Q  p.P95R | 2.37  1.06 |
| 105 | KIT | p.D816Y | 2.14 |
| 8 | NPM1 | p.L287fs | 2.32 |
| 193 | NRAS | p.G12D | 1.89 |
| 137 | TET2  ETV6 | p.R1465X  p.R105G | 7.49  2.31 |
| 279 | TET2 | p.G1564fs | 1.84 |
| 158 | TET2 | p.Q593X | 30.01 |
| 24 | TET2 | p.R1261S | 4.36 |
| 168 | U2AF1 | p. S34F | 1.59 |
| 78 | U2AF1 | p. S34F | 24.33^[[2]](#footnote-2)^ |
|  |  |  |  |

Fig.1


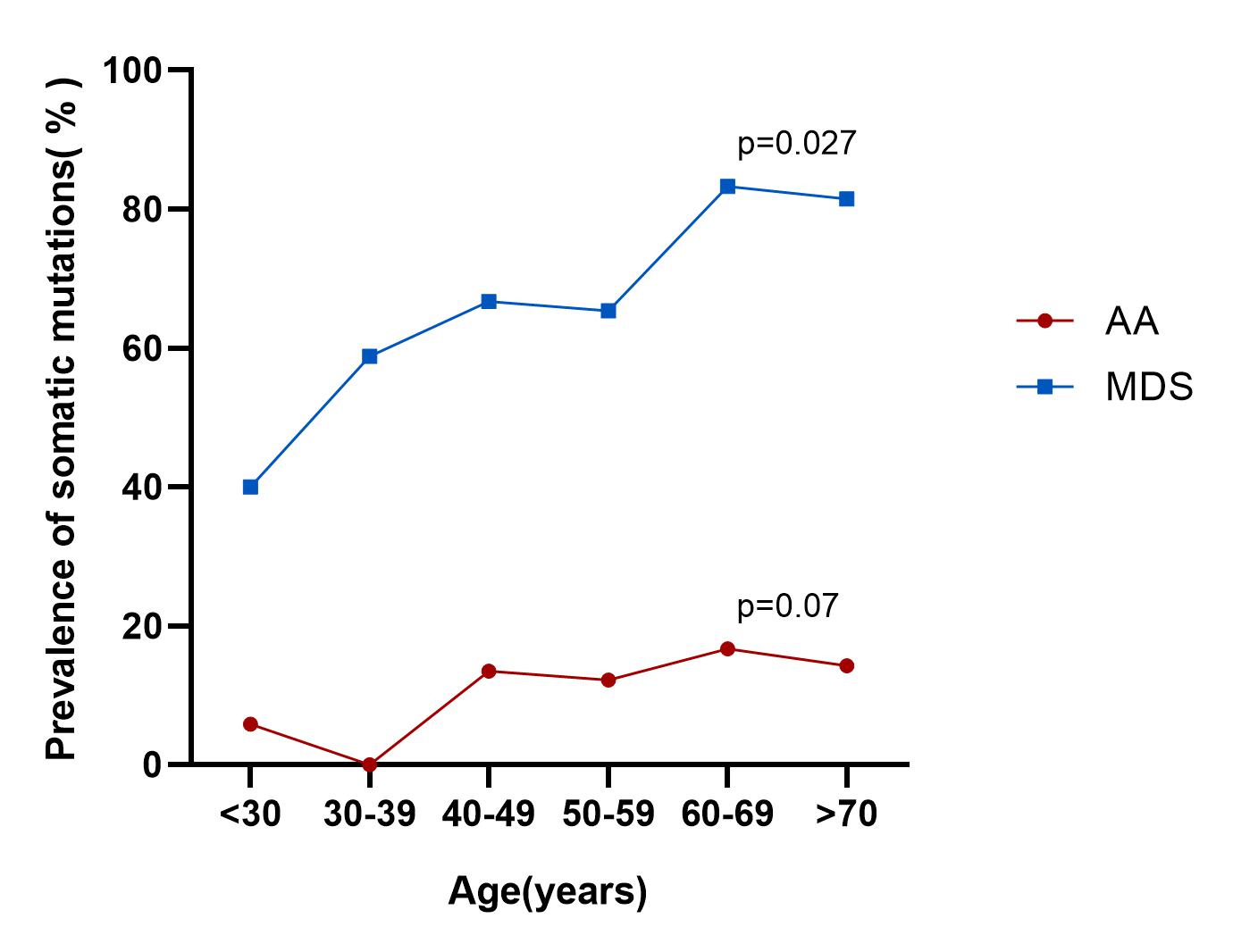


**Fig.1** The line plots represent the frequency of mutations in various age groups. The red line represents the AA patients(p=0.07), whereas the blue line represents the MDS patients, the proportion of mutation increased with age(p=0.027).

1. AA, aplastic anemia; MDS, myelodysplastic syndromes. [↑](#footnote-ref-1)
2. AA, aplastic anemia; VAF, variant allele frequency. [↑](#footnote-ref-2)
